# Reflective imaging of myelin integrity in the human and mouse central nervous systems

**DOI:** 10.3389/fncel.2024.1408182

**Published:** 2024-07-10

**Authors:** Georgina A. Craig, Lucy Ryan, Jessica Thapar, Niamh B. McNamara, Alana Hoffmann, Danielle Page, Jamie Rose, Simon R. Cox, Veronique E. Miron

**Affiliations:** ^1^Keenan Research Centre for Biomedical Science, St. Michael’s Hospital, Unity Health Toronto, Toronto, ON, Canada; ^2^United Kingdom Dementia Research Institute, The University of Edinburgh, Edinburgh, United Kingdom; ^3^Department of Immunology, The University of Toronto, Toronto, ON, Canada; ^4^Lothian Birth Cohorts, The University of Edinburgh, Edinburgh, United Kingdom

**Keywords:** myelin, reflectance, imaging, oligodendrocyte, compaction

## Abstract

The structural integrity of myelin sheaths in the central nervous system (CNS) is crucial for the maintenance of its function. Electron microscopy (EM) is the gold standard for visualizing individual myelin sheaths. However, the tissue processing involved can induce artifacts such as shearing of myelin, which can be difficult to distinguish from true myelin abnormalities. Spectral confocal reflectance (SCoRe) microscopy is an imaging technique that leverages the differential refractive indices of compacted CNS myelin in comparison to surrounding parenchyma to detect individual compact myelin internodes with reflected light, positioning SCoRe as a possible complementary method to EM to assess myelin integrity. Whether SCoRe is sensitive enough to detect losses in myelin compaction when myelin quantity is otherwise unaffected has not yet been directly tested. Here, we assess the capacity of SCoRe to detect differences in myelin compaction in two mouse models that exhibit a loss of myelin compaction without demyelination: microglia-deficient mice (*Csf1r*-*FIRE*^*Δ*/Δ^) and wild-type mice fed with the CSF1R inhibitor PLX5622. In addition, we compare the ability to detect compact myelin sheaths using SCoRe in fixed-frozen versus paraffin-embedded mouse tissue. Finally, we show that SCoRe can successfully detect individual sheaths in aged human paraffin-embedded samples of deep white matter regions. As such, we find SCoRe to be an attractive technique to investigate myelin integrity, with sufficient sensitivity to detect myelin ultrastructural abnormalities and the ability to perform equally well in tissue preserved using different methods.

## Introduction

The myelin sheath is a compact, multilamellar, lipid-rich membrane surrounding the axons of neurons in the peripheral and central nervous systems (PNS and CNS, respectively). Myelin sheaths insulate axons, enabling faster axonal conduction velocity and providing metabolic and trophic support to underlying neurons (Baumann and Pham-Dinh, 2001). If the structural integrity of myelin is compromised, CNS and axonal functions are jeopardized (Nave et al., 2023). Therefore, maintaining myelin integrity is increasingly recognized as paramount to ensuring neural health. Indeed, myelin ultrastructural irregularities in mice are associated with behavioral deficits (Gould et al., 2018; Arinrad et al., 2023; Groh et al., 2023; McNamara et al., 2023) and are sufficient to accelerate pathology in an Alzheimer’s disease mouse model (Depp et al., 2023). In humans, white matter abnormalities identified by magnetic resonance imaging are early indicators of poor cognitive prognosis in aging (Wardlaw et al., 2015). Myelin outfoldings—a type of ultrastructural abnormality—are corrected for by microglial engulfment during early development (Djannatian et al., 2023), but these myelin deficits re-emerge in aging (Safaiyan et al., 2016) and correlate with age-related cognitive impairment in non-human primates (Peters and Sethares, 2002). Along with the recent discoveries that oligodendrocytes—the myelinating cells of the CNS—become dysfunctional with age and disease (Kenigsbuch et al., 2022; Molina-Gonzalez et al., 2022), these data strongly position myelin integrity as a key predictor of functional decline in aging and disease in humans and animal models alike (Bartzokis, 2004; Nave et al., 2023).

However, confident quantification of myelin integrity remains an elusive task. The gold standard for visualization of myelin ultrastructure is electron microscopy (EM), which has an optical resolution powerful enough to visualize individual myelin wraps. When EM is used in the context of analyzing myelin integrity, it is important to note that conventional fixation methods do not preserve lipid-rich structures as well as lipid-poor structures (Möbius et al., 2016). This can lead to tissue fixation artifacts that resemble *bona fide* myelin abnormalities (Peters, 2002) and are exacerbated in experimental conditions where endogenous lipid levels are altered, such as in mice deficient in the myelin gene *Plp* (Klugmann et al., 1997). Relying solely on EM to interpret ultrastructural myelin deficits, therefore, runs the risk of misclassifying technical noise as a biological signal.

A complementary method to assess compact myelin is spectral confocal reflectance (SCoRe) microscopy. The SCoRe takes advantage of the high refractive index of myelinated tissue to detect compact myelin using reflected light (Schain et al., 2014). This relies on the principle that light hitting the surface of a thin film (in this instance, layers of lipid-rich myelin membrane), will reflect off this film and constructively and destructively interfere with other light waves, causing visible reflectance of light at some wavelengths but not others. Implementation of SCoRe thus involves shining lasers of different wavelengths upon a piece of myelinated tissue and capturing incident light reflected from the tissue back to the detector. Considering that each laser has a distinct wavelength, each laser will exhibit a unique pattern of constructive and destructive interference and thus will reflect a unique signature of myelin to the detector. For this reason, multiple channels representing reflectance from separate laser wavelengths are generally merged to create one cohesive SCoRe image (Schain et al., 2014). This can be done on a large scale and with conventional confocal systems, facilitating the ease and context in which SCoRe can assess compact myelin. Furthermore, unlike EM, SCoRe should not introduce artifacts that could be misconstrued as myelin irregularities. SCoRe has been used to track myelin deposition over murine development and aging (Hill et al., 2018), to quantify myelin loss in demyelinating injury (Gonsalvez et al., 2019), and as a label-free reporter of myelin for *in vivo* imaging studies (Auer et al., 2018; Hill et al., 2018; Chapman et al., 2023).

Despite its promise, SCoRe has remained a relatively underutilized technique in the myelin field. In part, this may be due to uncertainty regarding how to implement SCoRe, the experimental conditions that affect signal clarity, and whether SCoRe is qualitatively and/or quantitatively sensitive enough to changes in myelin ultrastructure to be comparable to EM analyses. To this latter point, it has been challenging to experimentally determine whether SCoRe reflects only compacted myelin (Gonsalvez et al., 2019). The production of a SCoRe signal depends on the phenomenon of thin-film interference, whereby light waves hit a thin layer, bounce on the upper and lower boundaries of this layer, and constructively and destructively interfere with one another to harmonically amplify certain light wavelengths and dampen others (Schain et al., 2014; Kwon et al., 2017). Thin-film interference requires the close apposition of several thin membranes, implying a threshold level of myelin compaction is needed to produce the SCoRe signal. However, where this threshold lies is unknown, and a loss of SCoRe signal may represent the loss of myelin compaction or the loss of myelin itself. Importantly, studies using SCoRe to investigate myelin integrity face the confounding issue that the experimental models used have features of myelin loss in addition to myelin integrity changes (Schain et al., 2014; Gonsalvez et al., 2019). As such, whether SCoRe can detect changes in myelin decompaction remains unclear.

In this paper, we investigate how well SCoRe performs at detecting myelin integrity loss without demyelination in the mouse CNS. We also quantitatively assess how well SCoRe performs following different tissue preservation strategies and under several microscopy conditions to inform the best use of this technique in future myelin studies. Finally, we successfully use SCoRe to visualize compacted myelin in paraffin-embedded human post-mortem samples, highlighting that this is a tractable technique to screen for myelin integrity in tissue samples that are unlikely to have been prepared for ultrastructural analyses. Together, the data presented herein highlight SCoRe as a readily employable technique for effectively quantitating myelin integrity in human and mouse CNS tissue.

## Materials and methods

### Animals

All animals used in this study were approved under project licenses approved by the UK Home Office and issued under the Animals (Scientific Procedures) Act. *Csf1r-FIRE*^Δ/Δ^ mice, wild-type controls from *Csf1rFIRE*^Δ/+^ crossings, and C57BL/6 J wild-type control mice were used. All animals were housed at a maximum of 6 animals per cage under 12 h light–dark cycles, with *ad libitum* access to food and drinking water. Both male and female *Csf1r-FIRE*^Δ/Δ^ animals were used for myelin integrity comparisons and for the experimental comparison between cryopreserved and paraffin-embedded tissue. The wild-type controls from *Csf1r-FIRE*^Δ/+^ crossings were fed with the CSF1R inhibitor PLX5622 (Chemgood, C-1521) in the chow at 1,200 ppm from 2 to 3 months of age, as previously shown to induce macrophage depletion and mimic the same myelin abnormalities seen in *Csf1r-FIRE*^Δ/Δ^ animals at 3–4 months of age (McNamara et al., 2023).

### Tissue collection and processing

All animals were anesthetized with 200 mg/mL of pentobarbital (Dolethal, Vetoquinol UK Ltd.) and, once vital reflexes had ceased, were intracardially perfused with 4% paraformaldehyde (PFA; Sigma), and their brains were extracted and post-fixed overnight in 4% PFA. For the analysis of the effects of cryo-embedding versus paraffin-embedding, the extracted brains were divided into two hemispheres. The right hemisphere was cryoprotected in 30% sucrose before being embedded in OCT (Tissue-Tech), snap-frozen on dry ice, and stored at −80°C prior to cryosectioning. Meanwhile, the left hemisphere was dehydrated in a series of graded Industrial Methylated Spirits (IMS) from 70 to 100%. The tissue blocks were then immersed in xylene and embedded in hot paraffin wax in sectioning cassettes before being cooled and stored at room temperature prior to sectioning. The cryo-embedded hemispheres were sectioned coronally with a cryostat at 10 μm thickness, while the paraffin-embedded hemispheres were sectioned coronally on a microtome at 10 μm thickness. The cryosections were thawed prior to SCoRe imaging, whereas the paraffin-embedded sections were dewaxed for 2 h at 60°C before being rehydrated in ethanol. Sections from each hemisphere were matched as best as possible to the same Bregma point in the corresponding hemisphere (range between approximately Bregma −0.10 and −0.96).

### Human post-mortem tissue

Human post-mortem tissue samples of healthy-aged human participants from the Lothian Birth Cohort 1936 (LBC1936) were obtained from the Medical Research Council Brain Bank at The University of Edinburgh (Research Ethics Committee (REC) approval 11/ES/0022). Post-mortem intervals ranged from approximately 1 to 4 days, with sampling undertaken according to a standard protocol (Henstridge et al., 2015). Access to tissue from the LBC1936 study was ethically obtained under a medical board peer review as part of an MRC Senior Non-Clinical Fellowship awarded to VEM, and the use of these samples was in accordance with the terms of use for donor tissue and information. Clinical history and neuropathologist examinations were provided by Prof. Colin Smith, University of Edinburgh. Formalin-fixed aged human tissue samples from a total of 16 cases, all from central white matter regions (Henstridge et al., 2015), were cut to 6 μm, deparaffinized, and imaged for SCoRe as described below.

### SCoRe imaging settings

All reflectance images of myelin were captured on a Leica-SP8 confocal microscope with an acoustic-optical beam splitter (AOBS) with a 70/30 reflectance/transmission (RT) ratio. The AOBS collected data from laser wavelengths of 488 nm, 568 nm, and 633 nm using three photodetectors (two HyD detectors and one PMT) with very narrow detection bandwidths centered around the chosen laser wavelengths. The minimum detection bandwidth was physically limited by the sliding mirrors to 5 μm, so detection bands were set to 485–490 nm, 565–570 nm, and 630–365 nm for each laser, respectively. Laser and gain settings were initially set at their minima and then slowly increased until the first over-saturated pixel appeared. At this point, the gain was slightly lowered until the first disappearance of the over-saturated pixel. For analyses that required fixed microscope settings across images, this process was executed for the first image and then laser/gain settings were kept the same for subsequent imaging. For the comparison of optimized settings, this process was repeated for every test subject and technical replicate. Two different immersion objectives were utilized in the process of benchmarking optimal SCoRe settings: a 40X water immersion objective (NA1.1) and a 40X oil immersion objective (NA1.25). In the case of the 40X water immersion objective for the benchmarking experiment murine sections were imaged prior to coverslip application, with a hydrophobic barrier drawn around each section and a generous amount of deionized water placed directly on top of the section as an immersion medium. Following this, slides were removed from the microscope, and mounting medium (Fluoromount G; Thermo Scientific 00–4,958-02) and coverslips (#1.5; Fischer Scientific; 12541033CA) were applied. The same sections and positions were then reimaged with water or oil lenses, using deionized water or oil as immersion media, depending on the lens type. For experiments where both a SCoRe image and a conventional fluorescence image were required in the same piece of tissue, this was achieved via the set-up of sequential scanning experiments on the Leica-SP8, with one sequence having a light path that captured reflected light and all other sequences being set to capture fluorescent light.

### SCoRe image analysis

For the quantitative analysis of the SCoRe signal, reflective images from each laser line/photodetector were merged into one composite image. Regions of interest were drawn around the cross-sectional area of the corpus callosum, or (in the case of the cortical murine analysis and human imaging), a rectangular region of interest of fixed measurement was placed in the area to be analyzed. A minimum threshold cutoff in FIJI (version 1.54) was then applied to identify those pixels positive for compact myelin. This generated a percentage of myelin compaction within these areas, as has been done previously (Gonsalvez et al., 2019; Govier-Cole et al., 2019). With the exception of the paraffin versus cryosection benchmarking experiment, for all analyses of SCoRe signal changes in murine images, at least three tiled images spanning the medial corpus callosum to the edge of the lateral ventricles were taken per animal with a 40X oil objective (NA1.1), and the SCoRe signal was averaged across three technical replicates. For human SCoRe imaging, at least four tiled (4×4) technical replicate images were taken per case per tissue sample, and the SCoRe signal was averaged across these images. For the paraffin versus cryosection benchmarking experiment, the same section was iteratively imaged across the four imaging conditions for a pairwise analysis of three animals.

### Immunohistochemical procedures

For immunostaining on human post-mortem tissue, paraffin-embedded sections were dewaxed for 2 h before being rehydrated in a graded ethanol series (from 100 to 70%). While membrane permeabilization is not ideal for the SCoRe signal, we found it necessary to permeabilize the membrane to some extent to enable staining within the human tissue. A 10-min submersion in methanol at −20°C was performed following ethanol rehydration, followed by 5 min of heat-induced epitope retrieval in sodium citrate buffer (pH = 6) at 90°C. The slides were then cooled gradually, first in a warm oven (60°C) for 30 min and then at room temperature. The sections were blocked in 10% normal horse serum with 0.5% Triton-X in PBS for 1 h, prior to the application of primary antibodies against rat-anti-MBP (1:250; Bio-Rad MCA409S), mouse-anti-CNPase (1:1000; Sigma AMAB91072), and chicken-anti-NFL-H (1:500; BioLegend 822,601) overnight at room temperature. The following day, the slides were washed three times in PBS for 5 min each before the application of secondary antibodies donkey-anti-rat-Alexa488 (1:200; Thermo Scientific A-21208), donkey-anti-mouse-Alexa568 (1:200; Thermo Scientific A10037), donkey-anti-chicken-Alexa647 (1:200; Thermo Scientific A78952), and Hoechst 33342 (1:10,000; Thermo Scientific H1399) for 2 h at room temperature. The slides were washed in PBS and mounted using Fluoromount G (Thermo Scientific 00–4,958-02) before coverslipping (#1.5; Fischer Scientific; 12541033CA).

### Data visualization

GraphPad Prism version 7 and BioRender were used to aid in data visualization.

## Results

### SCoRe imaging quantifies the loss of myelin integrity in the absence of demyelination

To investigate whether SCoRe imaging would be capable of quantifying differences in myelin integrity in a mouse model where myelin decompaction occurs but there is no loss of myelin, we utilized the *Csf1r*-*FIRE*^*Δ*/Δ^ mouse line in which the Fms intronic regulatory element (FIRE) super-enhancer of the CSF1R gene is removed (Rojo et al., 2019). This results in a loss of microglia in the CNS and an associated 25–40% of myelinated axons showing ‘abnormal’ profiles in early adulthood (1–4 months of age) in the corpus callosum (myelin sheaths with outfoldings, decompaction, and increased inner tongue areas) (McNamara et al., 2023). Mice at 3–4 months of age also display a hypermyelinating phenotype, whereby myelin is thicker; however, there is importantly no change in the density of myelinated axons either by EM or fluorescent staining for myelin proteins (McNamara et al., 2023). By 6 months of age, *Csf1r-FIRE*^*Δ*/Δ^ mice show signs of demyelination, and as such, animals beyond 4 months old were excluded from this study. In this way, changes in SCoRe signal in 1–4-month-old *Csf1r-FIRE*^*Δ*/Δ^ mice reflect solely losses in myelin integrity rather than a combination of myelin integrity loss and demyelination.

**Figure 1:**
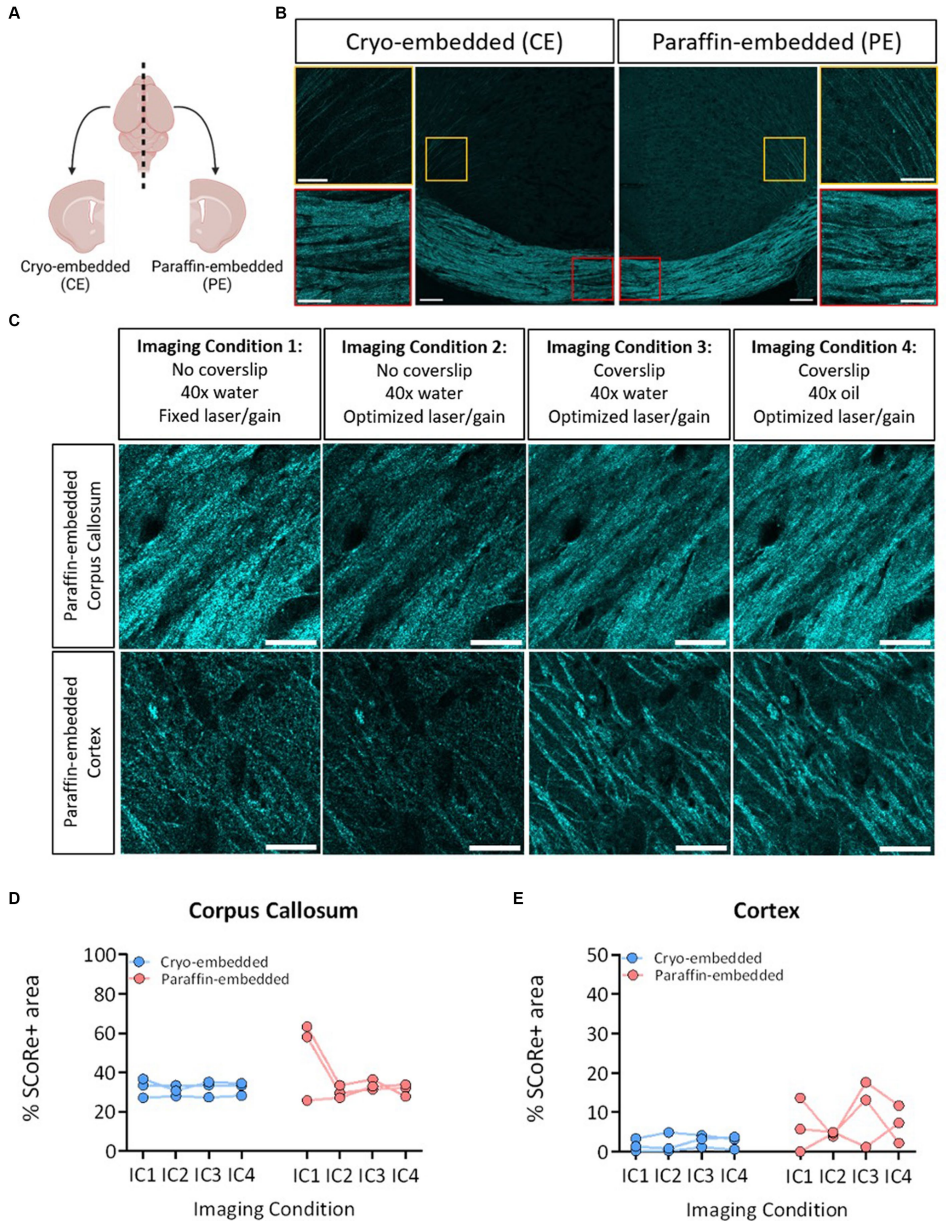
SCoRe imaging detects myelin in paraffin-embedded tissue. **(A)** Schematic of experimental workflow. After fixation, the left hemisphere from each animal (*n* = 3) was cryo-embedded, while the right hemisphere was paraffin-embedded, and sections from each hemisphere were cut to the same Bregma point. **(B)** Representative images of SCoRe signal in a cryo-embedded (left) versus paraffin-embedded (right) corpus callosum and cortex from the same animal, with zoomed-in panels highlighting individual myelinated fibers in the cortex (top images; yellow) and corpus callosum (bottom images; red). Scale bar 100 μm (top) and 20 μm (bottom). **(C)** Experimental workflow and representative images of the various imaging conditions (ICs) that each tissue section was subjected to, showing the same representative paraffin-embedded section in the corpus callosum (top panel) across ICs and the cortex (bottom panel) across ICs. The scale bar represents 50 μm. **(D)** Percentage area positive for SCoRe signal in the corpus callosum across tissue preservation type and ICs. There was no significant effect of preservation type (*p* = 0.3277), imaging condition (*p* = 0.1939), nor an interaction effect (*p* = 0.1863) when tested with a repeated-measures two-way ANOVA with a Geisser–Greenhouse correction. No individual comparisons were significant when testing for multiple comparisons with Tukey’s *post-hoc* correction. **(E)** Quantification of the percentage area positive for SCoRe signal in the corpus callosum across tissue preservation type and ICs. There was no significant effect of preservation type (*p* = 0.1158), imaging condition (*p* = 0.4162), or an interaction effect (*p* = 0.6857) when tested with a repeated-measures two-way ANOVA with a Geisser–Greenhouse correction. No individual comparisons were significant when testing for multiple comparisons with Tukey’s *post-hoc* correction. The dots represent individual animals, with lines connecting the same animal across ICs. Total *n* = 3 mice per group.

SCoRe imaging was performed in coronal sections of 1- and 3–4-month-old *Csf1r*-*FIRE*^*Δ*/Δ^ and wild-type littermate control mice, centering on the major white matter tract of the corpus callosum. Each wavelength of laser light hits the myelin sheath at a distinct angle and thus reflects in a unique pattern back to the detector (Schain et al., 2014; Gonsalvez et al., 2019). Three different laser wavelengths (488 nm, 561 nm, and 633 nm) were used to produce three reflective signals, which were then layered in one multichannel composite image (Figure 1A). The total myelinated area within the region of the corpus callosum was quantified as the percentage of area in the composite image with pixel intensity values above a fixed threshold. *Csf1r*-*FIRE*^*Δ*/Δ^ animals exhibited a 36.4% decrease in the percentage area positive for SCoRe signal in comparison to wild-type littermates (26.2% versus 41.2%) at both ages (Figure 1B)—largely comparable to the 40% increase in abnormally myelinated axonal profiles described in *Csf1r*-*FIRE*^*Δ*/Δ^ animals by EM (McNamara et al., 2023).

To understand whether these findings represented a reduction in myelin compaction versus a general reduction in myelin, a separate cohort of *Csf1r*-*FIRE*^*Δ*/Δ^ and wild-type littermate control mice were imaged for SCoRe both before and after myelin protein staining (Supplementary Figure S1A). The staining protocol itself did not have an impact on the SCoRe signal (Supplementary Figure S1B). Consistent with prior results of a myelin decompaction phenotype in *Csf1r*-*FIRE*^*Δ*/Δ^ mice without detectable changes in myelin protein levels by immunohistochemistry (McNamara et al., 2023), we found no relationship between CNPase or MBP protein intensity and relative SCoRe signal in *Csf1r*-*FIRE*^*Δ*/Δ^ mice (Supplementary Figures S1C,D).

To validate these findings in an alternate model of loss of myelin integrity in the absence of demyelination, we analyzed the SCoRe signal in wild-type mice fed the CSF1R inhibitor PLX5622 in the chow from 2 to 3 months of age. This treatment has been shown to induce myelin abnormalities that mimic those seen in 3-month-old *Csf1r*-*FIRE*^*Δ*/Δ^ mice without demyelination (McNamara et al., 2023). The area of the SCoRe signal was significantly reduced in PLX5622-treated animals compared to those on a normal diet, indicating a loss of compact myelin (Figures 1C,D).

### SCoRe imaging quantifies myelin in cryopreserved and paraffin-embedded tissue sections

Losses in myelin compaction have long been thought to drive cognitive decline in aging humans (Bartzokis, 2004). However, electron microscopic analyses of aged human white matter CNS tissue are exceedingly rare (Uranova et al., 2011; Liewald et al., 2014; Liu and Schumann, 2014), impeding histological validation of loss of myelin integrity with human age. Indeed, whether the white matter abnormalities identified by magnetic imaging-based studies truly reflect myelin irregularities is a matter of ongoing investigation (Wardlaw et al., 2015). Given SCoRe was able to detect myelin integrity differences in the *Csf1r*-*FIRE*^*Δ*/Δ^ mouse model consistent with previously reported EM results (McNamara et al., 2023), we next asked whether SCoRe would be a viable technique to quickly and easily screen for compact myelin density in aged human white matter without the need for specialized electron microscopic processing.

**Figure 2:**
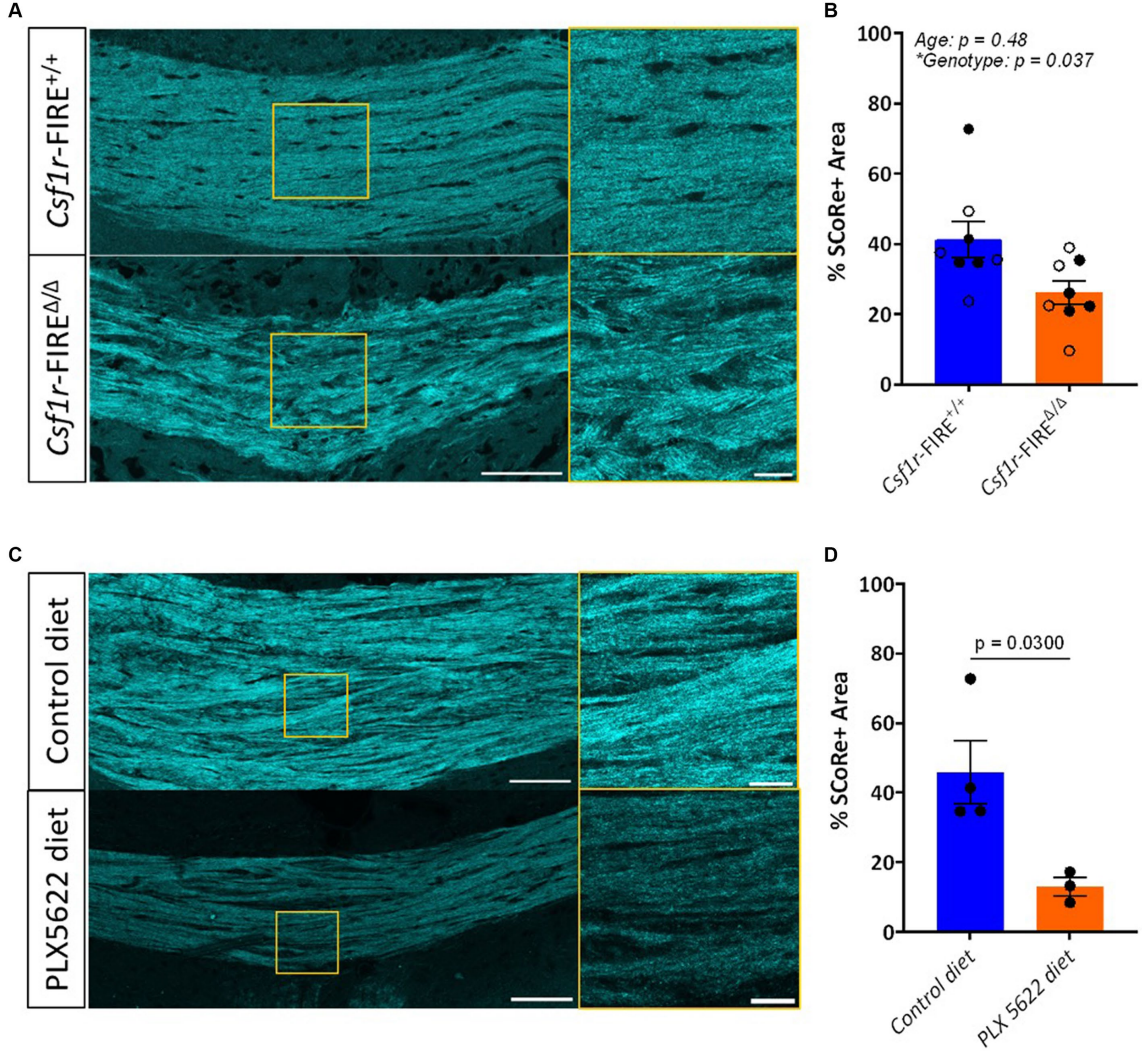
SCoRe signal is reduced in mice lacking myelin integrity. **(A)** Representative images of SCoRe signal in a tiled image of the corpus callosum of a 3-month wild-type (top) versus a 3-month *Csf1r*-FIRE^∆/∆^ (bottom) animal, with a zoomed inset panel outlined in yellow (right). Scale bar 100 μm (left) and 20 μm (right). **(B)** Average percentage area of the corpus callosum positive for SCoRe signal per animal ± S.E.M. Open circles represent animals aged 1 month, and closed circles represent animals aged 3 months. *n* = 4 animals per age per genotype, with the main effect of age and genotype calculated via two-way ANOVA. Tukey’s p*ost-hoc* comparison testing showed no significant within-age differences between genotypes. **(C)** Representative images of SCoRe signal in tiled images of the corpus callosum of 3-month wild-type mice with control diet (top) or PLX5622 diet (bottom) from 2 to 3 months, with a zoomed inset panel outlined in yellow (right). Scale bar 100 μm (left) and 20 μm (right). **(D)** Average percentage area of the corpus callosum positive for SCoRe signal per animal ± S.E.M. *n* = 3–4 animals per genotype, with differences between treatment types calculated with an unpaired Student’s *t*-test (*p* = 0.0300).

Considering human post-mortem tissue samples are often stored in paraffin, we first sought to understand whether paraffin-embedding would differentially impact the ability of SCoRe to detect myelin, which has to date only been performed in fresh (Schain et al., 2014; Auer et al., 2018; Hill et al., 2018; Chapman et al., 2023) or fixed-frozen (Schain et al., 2014; Gonsalvez et al., 2019) tissue. To benchmark the effect of preservation type, wild-type C57BL/6 J mice were collected such that one hemisphere of each animal’s brain was formalin-fixed and embedded in paraffin, and the other hemisphere was fixed and frozen (Figure 2A). The hemispheres were then sectioned with a microtome or cryostat, respectively, to the same bregma points. This enabled us to assess the SCoRe signal across the corpus callosum within the same mouse but with two distinct tissue preservation procedures (Figure 2B). Each hemispheric section was then iteratively reimaged, allowing for the quantification of the impact of tissue preservation type on fixed versus variable laser/gain settings, coverslip application, and immersion medium within the same animal (Figure 2C). Care was taken to image in the medial z-plane of the tissue sections, such that aberrant interference patterns from either the glass coverslip or microscope slide were minimized (Supplementary Figures S2A,B).

Composite images of the SCoRe signal were analyzed in either the medial corpus callosum or where cortical projection fibers radiate to the superficial cortical layers near the lateral peaks of the cingulate gyrus (Figure 2B). With the initial settings of water immersion, no coverslip, and fixed laser/gain settings across all three animals, the percentage area positive for SCoRe signal in the paraffin-embedded tissue was nearly double that of the cryo-embedded tissue (Figure 2D). When laser/gain settings were optimized for each animal, this variability was reduced, and the quantitation of the SCoRe signal in the corpus callosum was equal across hemispheres (Figure 2D). Keeping optimal microscopy settings, but applying a coverslip or changing the immersion medium did not result in quantitative differences in the SCoRe signal in the corpus callosum (Figure 2D).

As expected, the SCoRe signal was much reduced in the cortex compared to the corpus callosum, given the differential abundance of myelin (Figures 2B,C). However, there was a higher degree of what appeared to be parenchymal reflectance in the paraffin-embedded cortical region (Figure 2C), which made the quantitation of the SCoRe signal more variable in the paraffin-embedded hemisphere versus the cryo-embedded hemisphere (Figure 2E). While using a coverslip or an oil immersion lens appeared to increase the variability of the SCoRe signal in the cortex in paraffin-embedded tissue (Figure 2E), this was not significant, and there was no effect of preservation type or imaging conditions on the SCoRe signal.

### SCoRe imaging detects compact myelin in aged human CNS white matter

**Figure 3:**
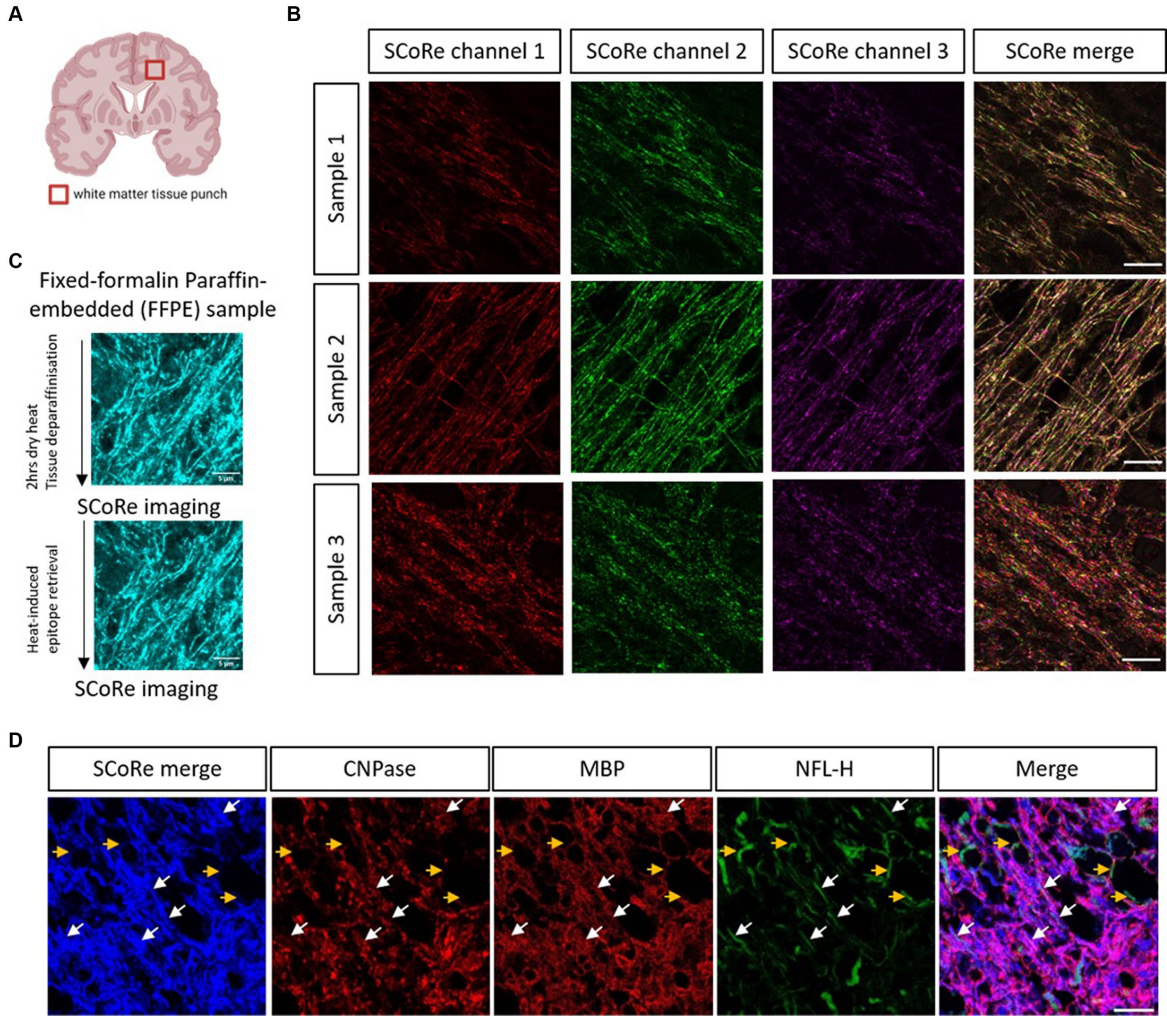
SCoRe imaging detects myelinated axons in aged human central white matter tissue. **(A)** Schematic of location of human brain tissue-punch of deep white matter. **(B)** SCoRe images of deep white matter in human CNS paraffin-embedded tissue across three samples, representing endogenous variability in myelin quality in aged human tissue. Different lasers reflect slightly different SCoRe patterns, which can be merged into a cohesive image. **(C)** Representative images of human central white matter imaged after deparaffinization but before heat-induced epitope retrieval (HIER) (top) versus after HIER (bottom) within the same tissue section. **(D)** The white arrows show SCoRe signal in human tissue (blue) which overlaps with CNP/MBP (red), and neurofilament heavy chain (green), indicating compacted myelin segments in human-aged white matter. The yellow arrows show unmyelinated, neurofilament-positive but SCoRe and MBP/CNP-negative, axonal segments, indicating that SCoRe signal is specific to myelinated portions of axons. The scale bar represents 10 μm.

Having established that SCoRe imaging detects compact myelin equally well in paraffin-embedded versus cryo-embedded samples, we then sought to understand whether SCoRe imaging could detect myelin in deep white matter regions of the human CNS from aged, healthy samples that had been paraffin-embedded and stored for several years (Figure 3A). The samples were dewaxed with heat and rehydrated in graded ethanol before being either immediately imaged for reflectance only (Figure 3B) or subjected to heat-induced epitope retrieval and stained with human-reactive antibodies. While the SCoRe signal varied considerably across separate human samples, we did not find that the heat retrieval qualitatively altered the SCoRe signal (Figure 3C), nor did the post-mortem interval have a significant effect (Supplementary Figure S3).

SCoRe imaging in the aged human tissue produced a stereotypical reflectance pattern, with each laser reflecting a slightly different striated pattern of myelin (Figure 3B), as expected for variable internode thickness along individual myelinated CNS fibers (Schain et al., 2014; Tomassy et al., 2014). To understand whether the reflective patterning seen was specific to myelinated internodes, the tissue was stained with two myelin markers, CNPase and MBP, in addition to the axonal marker neurofilament. We found several instances of neurofilament-positive axons that were negative for both myelin markers and the merged SCoRe signal, confirming that non-myelinated fibers in human-aged white matter do not produce a reflective signal (Figure 3D). Notably, there were numerous instances where the SCoRe signal overlapped with both myelin proteins and neurofilament, indicating compacted myelin (Figure 3D). Instances where myelin protein is present, and the SCoRe signal is absent should indicate areas of decompacted myelin, as previously shown by live imaging of early degenerating myelin sheaths (Chapman et al., 2023). Together, these data show that SCoRe imaging can be successfully applied to human paraffin-embedded samples to detect compact myelin. This will be a useful technique to screen human tissue for compact myelin in situations where typical antibody labeling proves difficult.

## Discussion

Myelin integrity is becoming increasingly recognized as having crucial importance for dictating neural health, particularly in aging and cognition (Bartzokis, 2004; Nave et al., 2023). However, the field lacks tools to assess myelin integrity in a cost-effective, bias-free manner on a large scale. Here, we benchmarked SCoRe imaging as a cheap, effective technique to quantify myelin integrity changes that can be applied to a wide variety of tissue types.

### SCoRe imaging is an effective technique for screening for myelin integrity

There are several techniques commonly used to assess the volume and integrity of myelin, including but not limited to dye-based methods (e.g., Luxol fast blue, Sudan dyes, and fluoro-myelin), immunohistochemistry for myelin proteins, image-based methods of analyzing directionality of water flow (e.g., DTI-MRI), second/third generation harmonic imaging, and EM (either with aldehyde-based fixatives or via high-pressure freezing and freeze-substitution). Of these, the only technique capable of visualizing myelin at the ultrastructural level is EM. In this paper, we asked how effective SCoRe would be in detecting compact myelin differences in white matter in mouse models previously shown to have myelin abnormalities by EM (*Csf1r*-*FIRE*^∆/∆^ mice, and wild-type mice treated with the CSF1R inhibitor PLX5622 from 2 to 3 months of age) (McNamara et al., 2023). Importantly, there are no signs of demyelination in these models at the ages used (McNamara et al., 2023), and the total quantity of myelin in the corpus callosum is equal across control and experimental groups. Therefore, it is likely that the decrease observed in the SCoRe signal in these models is attributable primarily to the loss of compact myelin ultrastructure rather than the loss of myelin. While previous studies have shown SCoRe imaging is sensitive to a loss of myelin integrity concurrent with demyelination, we assess SCoRe imaging as being sufficiently sensitive to detect myelin integrity changes without demyelination.

Importantly, when the level of myelin decompaction previously reported in the *Csf1r*-FIRE^∆/∆^ mouse using EM (McNamara et al., 2023) was compared to the percentage area of SCoRe signal for *Csf1r*-FIRE^∆/∆^ mice, we found that both techniques showed a reduction in compacted myelin. Previous analyses by EM showed that the percentage of abnormally myelinated fibers in the corpus callosum of young adult *Csf1r*-FIRE^∆/∆^ mice is ~45%, compared to 5% in wild-type littermates (McNamara et al., 2023). Consistent with this, we find that the percentage of corpus callosum area positive for the SCoRe signal decreases from a mean of 41.2% in the wildtype to a mean of 26.2% in the FIRE animals—a 36.4% decrease when normalized to the SCoRe signal in the wildtype. Therefore, using either EM or SCoRe shows that ~35–40% of the myelinated axons present in the *Csf1r*-FIRE^∆/∆^ mice are likely to have a dysmyelinated profile.

Previously, SCoRe has been used to identify compact myelin (Schain et al., 2014; Auer et al., 2018; Chapman et al., 2023), measure developmental myelination (Hill et al., 2018), or quantify compact myelin loss during demyelination (Gonsalvez et al., 2019). This study adds to the contexts in which SCoRe can be implemented by showing it is also sufficient to detect changes in myelin integrity. This study therefore highlights that changes in SCoRe may be indicative not only of demyelination but general myelin sheath decompaction. As such, further studies seeing myelin change using SCoRe would be best placed to use EM as a complementary technique to discern which of these myelin changes (decompaction or demyelination) might underlie any phenotypes observed.

However, while SCoRe complements rather than replaces the need for detailed electron microscopic analysis of myelin, it does offer several benefits over the latter technique: it is much faster, less labor-intensive, and capable of canvassing a much larger area than EM. Furthermore, as SCoRe can be used *in vivo,* this opens up the possibility of detecting changes in myelin compaction in real time. This will be particularly useful in the contexts of learning, aging, or neurodegeneration, where the deposition or loss of compact myelin is known to play a role. White matter changes have been identified as early hallmarks in a number of neurodegenerative conditions (Davis et al., 2003; Uranova et al., 2011; Philips et al., 2013; Agosta et al., 2014; Brickman et al., 2015; Mathys et al., 2019; Chen et al., 2020, 2021; Sadick et al., 2022), and using SCoRe to understand the degree to which myelin compaction may have changed in these contexts will be a critical step toward understanding the pathology of such diseases.

### SCoRe can be used on CNS tissue of different preservation types

Studies using human tissue often face the hurdle that samples are embedded in paraffin; considering that the deparaffinization process requires the use of solvents that can extract lipids (Kiernan, 2007), whether paraffin-embedded tissue would be suitable for SCoRe was hitherto unknown. To our knowledge, there have been no instances of SCoRe use in paraffin-embedded tissue, with its uses being limited to fresh (Schain et al., 2014; Auer et al., 2018; Hill et al., 2018; Chapman et al., 2023), fresh-frozen (Schain et al., 2014), or fixed-frozen (Gonsalvez et al., 2019) tissue.

We found that cryo-embedding produced a more consistent SCoRe signal than paraffin-embedding, especially for myelin imaging in the cortex. However, the two techniques were largely equivalent, particularly if water immersion dipping objectives were used without a coverslip. The increased background reflectance signal seen in the paraffin-embedded cortex may be due to incomplete dewaxing or rehydration during the deparaffinization process, and care should be taken to thoroughly deparaffinize with fresh solvents and alcohols to limit any background reflectance in future SCoRe studies. However, paraffin-embedding does not present an irrevocable hurdle to assessing myelin integrity with SCoRe imaging, and we hope that this comparison paves the way forward for researchers looking to assess myelin integrity changes in paraffin-embedded samples.

## Conclusion

In summary, we find that SCoRe imaging provides an accessible technique to quickly and easily screen for myelin integrity changes in animal and human CNS tissue preserved using standard approaches. We hope that exploring the contexts in which this technique can be adopted will make a tangible difference to the ease with which myelin researchers uptake this exciting technology.

## Data availability statement

The raw data supporting the conclusions of this article will be made available by the authors, without undue reservation.

## Ethics statement

The studies involving humans and animals were approved by the Medical Research Council Research Ethics Committee at the University of Edinburgh. The studies were conducted in accordance with the local legislation and institutional requirements. The participants provided their written informed consent to participate in this study.

## Author contributions

GC: Writing – review & editing, Writing – original draft, Visualization, Methodology, Formal analysis, Data curation, Conceptualization. LR: Writing – review & editing, Formal analysis, Conceptualization. JT: Writing – review & editing, Data curation. NM: Writing – review & editing, Resources. DP: Writing – review & editing, Resources. JR: Writing – review & editing. SC: Writing – review & editing, Resources, Funding acquisition. VM: Writing – review & editing, Supervision, Resources, Project administration, Funding acquisition. AH: Resources, Writing – review & editing.
