# Supplementary material for: Reflective imaging of myelin integrity in the human and mouse central nervous systems
Source: Front Cell Neurosci. 2024 Jul 10;18:1408182. doi: 10.3389/fncel.2024.1408182 (PMC11266064; doi:10.3389/fncel.2024.1408182)
Supplement: Supplementary file 1 [file Data_Sheet_1.docx]

**Supplementary Figure 1. SCoRe signal is not correlated with myelin protein staining**

**
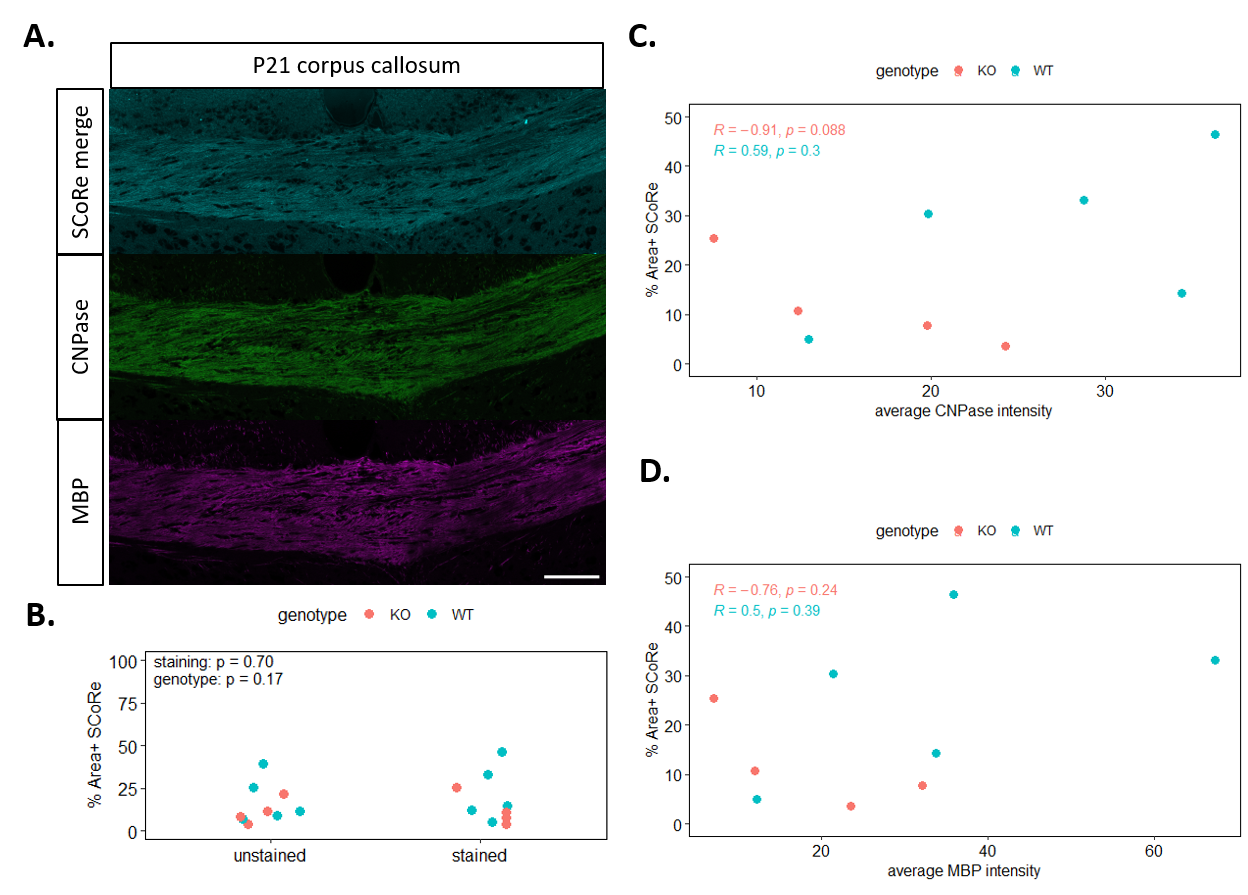
**

**A.** Representative images of a P21 corpus callosum wildtype animal stained with MBP, CNPase and also imaged for SCoRe signal (merge of lasers 488, 561, and 633nm). Scale bar represents 100µm. **B.** Antigen retrieval associated with the staining protocol does not quantitatively alter SCoRe signal in P21 wildtype nor P21 *Csf1r*-FIRE^∆/∆^ animals. Effect of staining and genotype assessed via Two-Way ANOVA. **C, D.** No correlation between the percentage of area positive for SCoRe signal above a fixed threshold in the corpus callosum of P21 wildtype or *Csf1r*-FIRE^∆/∆^ animals and intensity of myelin protein staining: CNPase (C) or MBP (D).


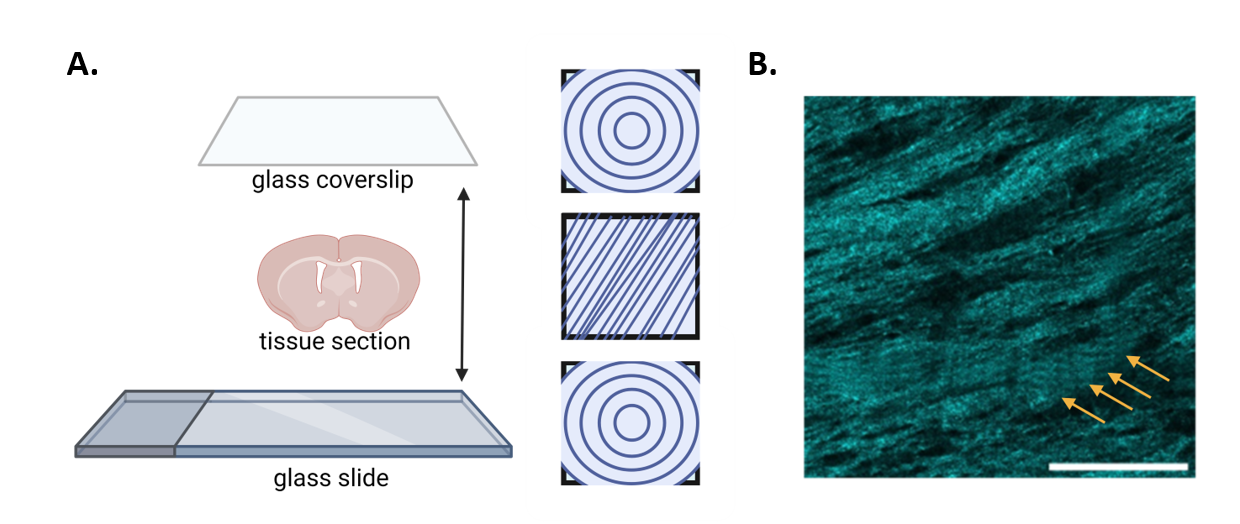
**Supplementary Figure 2. Myelin aberrations can occur if imaging too close to the coverslip**

**A.** Schematic showing expected circular image aberrations which can occur when imaging reflected light too close to the glass surface of the coverslip or slide (top and bottom images), as opposed to expected striatal SCoRe pattern (middle image). **B.** Representative image of SCoRe aberration in murine corpus callosum. Scale bar represents 50µm.


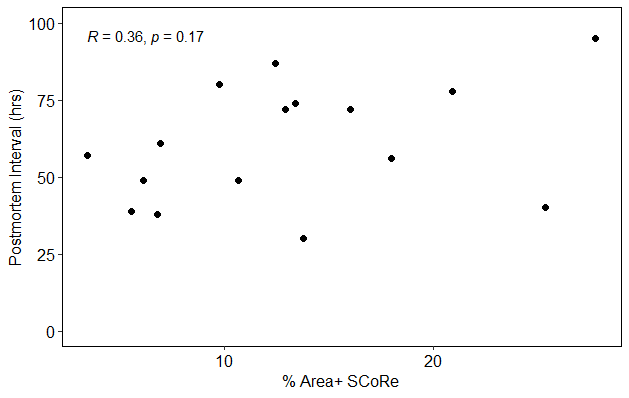
**Supplementary Figure 3. Human tissue quality is not associated with SCoRe signal**

The percentage of area positive for SCoRe signal in deep human white matter FFPE sections is not correlated to the post-mortem interval (hrs) of the tissue.
